# Accelerating the Measurement of Fatigue Crack Growth with Incremental Information-Based Machine Learning Approach

**DOI:** 10.3390/ma19020396

**Published:** 2026-01-19

**Authors:** Cheng Wen, Haipeng Lu, Yiliang Wang, Meng Wang, Yuwan Tian, Danmei Wu, Yupeng Diao, Jiezhen Hu, Zhiming Zhang

**Affiliations:** 1 School of Mechanical Engineering, Guangdong Ocean University, Zhanjiang 524088, China; 2 Institute of Corrosion Science and Technology, Guangzhou 510530, China; 3 Zhanjiang Key Laboratory of Ocean Engineering and Equipment Corrosion and Protection, Guangdong Ocean University, Zhanjiang 524088, China; 4 Guangdong Provincial Ocean Equipment and Manufacturing Engineering Technology Research Center, Guangdong Ocean University, Zhanjiang 524088, China

**Keywords:** fatigue crack growth, incremental information, machine learning, extrapolation prediction

## Abstract

Measuring the fatigue crack growth rate via the crack growth experiment (*a*-*N* curve) is labor-intensive and time-consuming. A machine learning interpolation–extrapolation strategy (MLIES) aimed at enhancing the prediction accuracy of small-data models has been proposed to accelerate fatigue testing. Two specific approaches are designed by transforming *a*-*N* curve data from *N* to *ΔN* and from *a* to *Δa* (S1)/*Δa*/*ΔN* (S2) to enrich the data volume and leverage the incremental information. Thus, a simple and fast-responding single-layer neural network model can be trained based on the early-stage data points from fatigue testing and accurately predict the remaining part of an *a*-*N* curve, thereby enhancing the experimental efficiency. Through exponential data expansion and data augmentation, the trained neural network model is able to learn the underlying rules governing crack growth directly from the experimental data, requiring no explicit analytical crack growth laws. The proposed MLIES was validated on fatigue tests for aluminum alloy and titanium alloy samples under different experimental parameters. Results demonstrate its effectiveness in reducing testing time/cost by 15–32% while achieving over 30% higher prediction accuracy for the *a*-*N* curve compared to a traditional machine learning modeling approach. Our research offers a data-driven recipe for accurate crack growth prediction and accelerated fatigue testing.

## 1. Introduction

Fatigue failure of metal components under alternating loads is a critical issue affecting structural safety and reliability. Fatigue crack growth (FCG) analysis based on the crack length–number of fatigue cycles relationship (i.e., *a*-*N* curve), which yields material properties such as the FCG rate, is an essential step for assessing structural remaining life and performing damage tolerance design. However, conventional experimental testing for *a*-*N* curves is limited by long cycle times and high costs. For decades, researchers have developed various methods to predict fatigue crack length. Early approaches primarily used formula-based fitting to describe how crack length evolves with cycle count. This led to the proposal of predictive models based on polynomial [1], exponential [2,3,4,5,6], and power function equations [7,8,9,10], among others, for generating *a*-*N* curves. Although more complex formulas may enhance prediction accuracy, the fitting approach itself is heavily reliant on human expertise and exhibits limited generalizability across broader applications.

In recent years, the rise of artificial intelligence (AI) has led to the development of numerous data-driven ML models for predictive analytics in fatigue-related engineering [11], such as fatigue life prediction [12,13], fatigue crack growth [14,15], fatigue damage diagnosis [16,17] and fatigue strength prediction [18,19], among which neural network-based fatigue life prediction is the most widely studied topic [20]. A common practice is to employ material and experimental variables as input variables and the fatigue life as the output. As for FCG prediction, usually, the stress intensity range *ΔK* is used as one of the inputs to ML modeling, and the output is the crack growth rate d*a*/d*N*. Depending on the problem to be investigated, other influencing factors are also included in the inputs, such as stress ratio *R*, crack length *a*, stress intensity factor *K*, and temperature *T* [21,22,23,24,25,26,27]. Although an *a*-*N* curve can be calculated via integration of d*a*/d*N*, the error will be cumulated during integration process. For example, Zhang et al. [28] and Mohanty et al. [29] employed RBF-ANN and MLP models, respectively, to predict FCG rates in aluminum alloys with good precision. While both studies employed integration to derive *a*-*N* curves, the predictions ultimately showed deviations from experimental data.

In fact, the crack length can also be modeled directly through the *a*-*N* relationship without detailed crack growth analysis. Pidaparti et al. [30] employed a feedforward neural network (FNN) to model the relationship between crack length and the number of fatigue cycles, in which the crack length served as the input to predict the corresponding cycle number. The predictions from this model showed an error of approximately 10% when compared to experimental data. Okafor et al. [31] investigated the use of acoustic emission (AE) for fatigue crack length prediction. A cascade FNN model was employed, using both the AE signal and the number of cycles as inputs to predict crack length as the output. The model, trained on 57 data points, demonstrated good predictive performance. Beyond the interpolation prediction, ML can be effectively explored for the extrapolation of crack length. An important application scenario is the online monitoring of in-service equipment for safety assurance. Nechval et al. [32] leveraged monitoring data and employed ML to model the relationship between *N* and *a*, thereby facilitating the construction of a framework for identifying non-steady-state crack propagation during fatigue service. Haynes et al. [33] embedded a neural network (NN) model within a structural health monitoring system to achieve precise prediction of fatigue long cracks in Aluminum 7075-T6 rivet holes, thereby guiding fatigue damage diagnosis. To date, there has been relatively limited research on predicting the *a*-*N* curve with the explicit aim of accelerating its measurement. This is primarily due to the limited number of data samples available for ML modeling, leading to inaccurate and unstable prediction results. Ma et al. [34] developed an FNN model that extrapolates the *a*-*N* curve from its initial segment, using the initial loading cycles and cycle increment as inputs to predict crack length and growth. The incremental learning scheme allows the model to outperform simple RNN and LSTM networks in prediction accuracy. However, the dual-input and dual-output structure complicates the network architecture, thereby increasing its training time. As reported, the total training time of 30,000 epochs based on their NN model is roughly 4.48 h on a server equipped with a 3.2 GHz Intel Core i7 when the number of trainable data on *a*-*N* curve is 55. Therefore, it functions more as an offline prediction method and cannot meet the requirement for real-time *a*-*N* curve testing based on online monitoring and responsive modeling.

**Figure 1:**
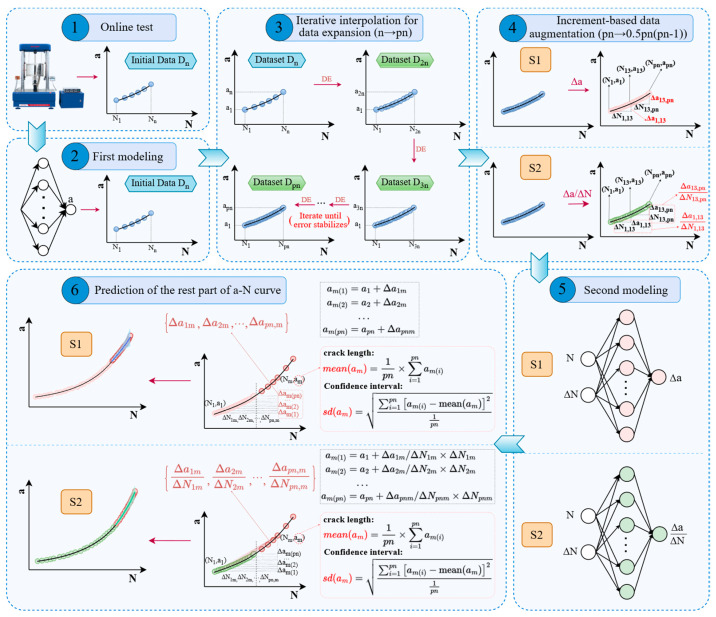
The workflow of the MLIES framework for accelerated prediction of fatigue crack length.

To overcome the limitations of traditional prediction methods and address the challenge of rapidly modeling and accurately predicting fatigue crack length using a small data sample, we developed a machine learning interpolation–extrapolation strategy (MLIES) utilizing incremental information to rapidly obtain complete *a*-*N* curves by leveraging limited early-stage online test data. As shown in Figure 1, the process begins with a first-phase feedforward neural network (FNN) model that takes cycle count (*N*) as input and predicts crack length (*a*). This model is then updated to iteratively expand the data set through interpolation, i.e., data expansion (DE). Next, the generated *a*-*N* data is converted, in an incremental manner, i.e., data augmentation (DA), into *Δa*-*ΔN* data (absolute increments-based data augmentation) and *Δa*/*ΔN*-*ΔN* data (relative increments-based data augmentation). This enriched incremental data, which contains derivative information, serves as the basis for constructing two subsequent models based on a single-layer FNN structure: Model S1 predicts *Δa* using *N* and *ΔN* as inputs, while Model S2 predicts *Δa*/*ΔN*. Finally, fatigue crack length, along with its confidence intervals, can be estimated from the ensemble prediction results. This allows for the extrapolation of the remaining portion of the *a*-*N* curve. The proposed MLIES effectively leverages limited online testing data. It achieves accurate extrapolation predictions via interpolation-based data expansion and increment-based data augmentation, thereby accelerating *a*-*N* curve measurement. Validation on aluminum and titanium alloys under various experimental parameters confirms its effectiveness. Compared to traditional methods, the MLIES improves prediction accuracy by over 30% and reduces testing time/cost by up to 32%.

## 2. Machine Learning Interpolation–Extrapolation Strategy

To enable rapid ML modeling and prediction, and to accommodate the nonlinear fitting required in later-stage *a*-*N* testing, our strategy avoids complex architectures. Instead, a single-hidden-layer network was employed. Regarding the number of nodes in the hidden layer, preliminary tests indicated that for small-sample data of an *a*-*N* curve, increasing the number beyond 15 does not yield significant improvements in prediction accuracy but does increase training time. Therefore, we employed a lightweight neural network with fewer than 15 neurons to achieve both rapid modeling and accurate prediction. All the codes were executed using R software (Version 4.4.0). Beginning with initial *a*-*N* data points (D_n_), as depicted in step 3 in Figure 1, an iterative modeling process was applied for interpolation prediction to expand data samples. The specific workflow is as follows:

Based on the dataset D_n_ from an online test, containing n sample points: {(*a*_1_, *N*_1_), (*a*_2_, *N*_2_), …, (*a*_n_, *N*_n_)}, the first-phase FNN model with N as input and a as output is built, and the associated prediction error can be determined. Subsequently, the cycle interval *N*_1_~*N*_n_ is divided into *p* equal intervals (*p* = 2) to obtain the corresponding cycles *N*_1_~*N*_2n_. The corresponding fatigue crack lengths *a*_1_~*a*_2n_ can be obtained through the model prediction. Then, based on the expanded dataset D_2n_, the FNN model is updated and simultaneously employed to predict the crack length of the D_n_ dataset, yielding the corresponding prediction error. By sequentially increasing the *p*-value (3, 4, 5...) to iteratively expand the dataset and calculating the prediction error until it converges, the optimal expansion factor can be determined. Accordingly, the iterative loop will yield an expanded dataset D_pn_: {(*a*_1_, *N*_1_), (*a*_2_, *N*_2_), ..., (*a*_pn_, *N*_pn_)}, as illustrated in Figure 1. DE enriches the base dataset, enabling full utilization of early-stage testing samples information.

**Figure 2:**
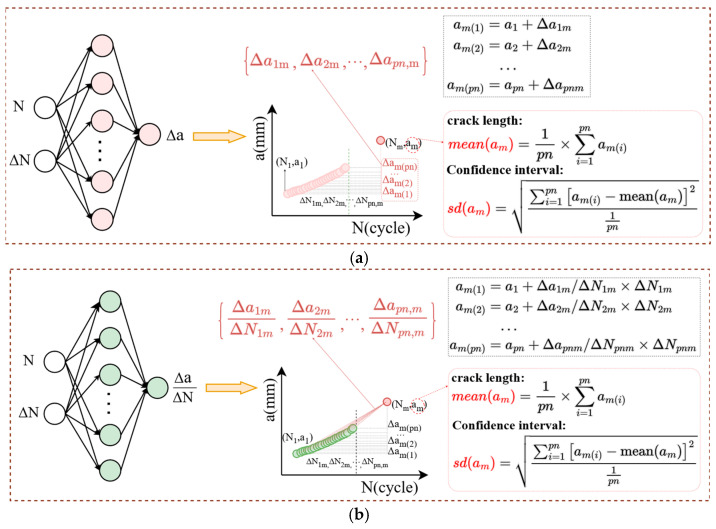
Fatigue crack length prediction strategy. (**a**) Absolute increments-based data augmentation and FNN modeling and prediction (S1); (**b**) Relative increments-based data augmentation and FNN modeling and prediction (S2).

Then, two DA approaches and ML modeling strategies based on incremental information were used, to enable the extrapolation prediction of late-stage crack length on the *a*-*N* curve. These are designated as S1 and S2, as shown in Figure 2.

S1 (absolute increments-based data augmentation): For data samples in D_pn_, the cycle intervals *ΔN*_ij_ (i < j) between each sample point and its corresponding crack length difference *Δa*_ij_ are calculated. This conversion transforms the *a*-*N* data into *Δa*-*ΔN* data, thus achieving an exponential increase in the number of data samples. Subsequently, a FNN model is trained using the cycle count *N*_i_ and cycle count interval *ΔN*_ij_ as dual inputs, with *Δa*_ij_ as the output. This establishes the mapping relationship *Δa*_ij_ = *f* (*N*_i_, *ΔN*_ij_). Consequently, the crack length *a*_m_ for any unknown cycle count *N*_m_ can be determined through model prediction, based on its association with the corresponding *p_n_* expanded sample points (*ΔN*_pn,m_), as illustrated in Figure 2a.

S2 (relative increments-based data augmentation): Unlike S1, which focuses solely on absolute crack length increments, the S2 further incorporates crack growth gradient increment to enhance the model’s extrapolation performance. Specifically, for data samples in D_pn_, the cycle intervals *ΔN*_ij_ (i < j) between each sample point and its corresponding crack growth rate *Δa*_ij_/*ΔN*_ij_ are calculated. This conversion transforms the *a*-*N* data into *Δa*/*ΔN*-*ΔN* data. Subsequently, by modeling the relationship *Δa*_ij_/*ΔN*_ij_ = *f* (*N*_i_, *ΔN*_ij_) using FNN, the crack length *a*_m_ for any unknown cycle count *N*_m_ can be determined through model prediction, based on its association with the corresponding *p_n_* expanded sample points (*Δa*_pn,m_/*ΔN*_pn,m_), as illustrated in Figure 2b.

It should be noted that our work aims to enhance data utility by fully uncovering the implicit physical information within small datasets for *a*-*N* curves. The approach of constructing and modeling incremental information data helps prevent possible large prediction deviations caused by overfitting in small-data models. However, the exponential increase in dataset size through incremental pairing does not correspond to a proportional increase in independent information, since the augmented samples originate from the same experimental data. Here, the incremental processing of data not only introduces additional latent physical information but also, in effect, modifies the data distribution and structure to some extent, thereby positively influencing the model’s predictions.

## 3. Results and Discussion

**Figure 3:**
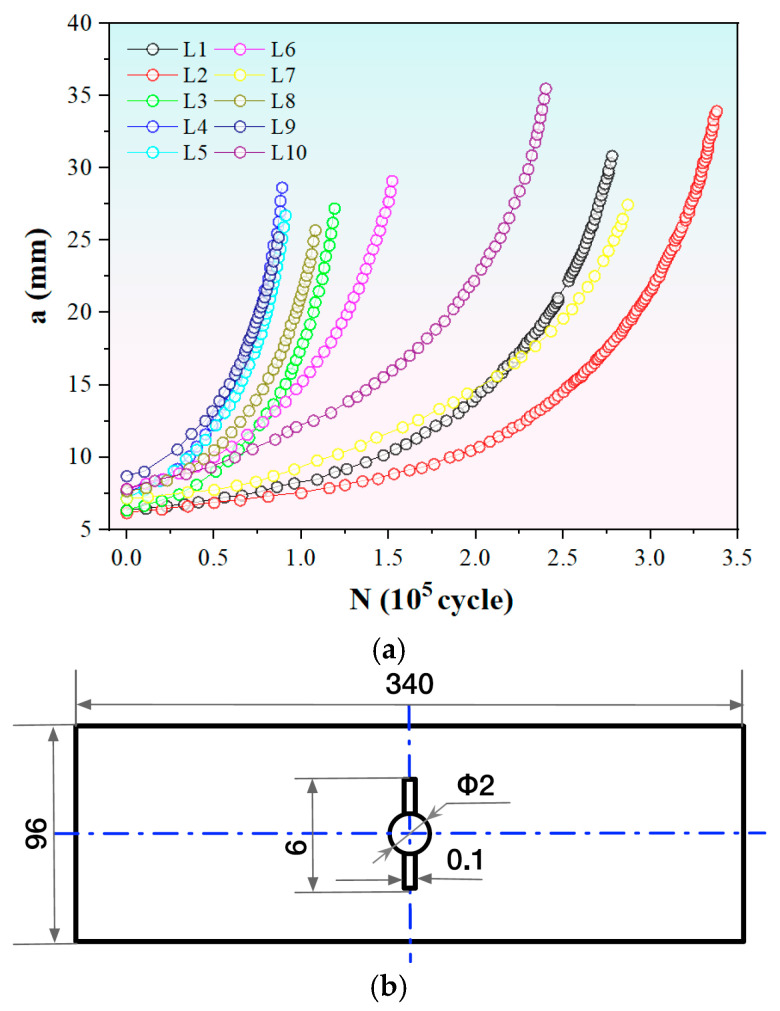
Data of *a*-*N* curves. (**a**) Fatigue curves measured under different experimental parameters. (**b**) The geometry of specimens used to obtain the fatigue curves. The unit is mm.

To validate the effectiveness of the proposed MLIES in accelerated fatigue curve testing, ten *a*-*N* curve datasets from middle-tension specimens for 7B04-T6 aluminum and TA15 titanium alloys were collected [34], as shown in Figure 3. Each curve was measured under distinct stress ratios, stress amplitudes, and initial crack lengths, with specific details presented in Table 1. It is evident that the crack propagation patterns vary significantly across different testing conditions, and the data sizes also exhibit considerable differences.

### 3.1. Data Expansion and Data Augmentation

**Figure 4:**
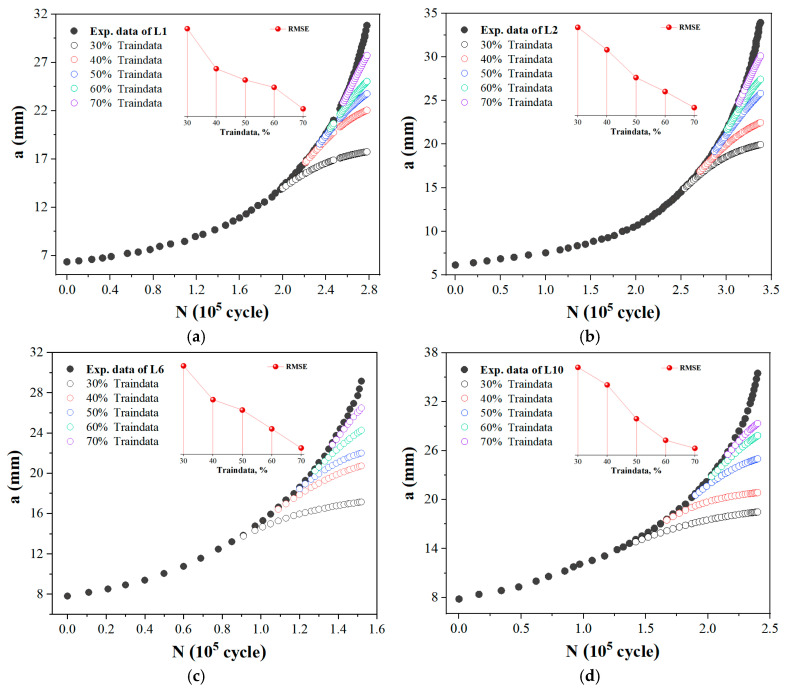
Fatigue crack length prediction by traditional single-input–single-output modeling method (T0). (**a**) L1; (**b**) L2; (**c**) L6; (**d**) L10.

In order to evaluate the predictive performance of conventional methods, four representative *a*-*N* curves of L1, L2, L6, and L10 were selected. Using *N* as the input and *a* as the output, the FNN models with different proportions of data points from these curves were trained. These models were then used to perform extrapolation predictions on the remaining portions of the *a*-*N* curves. From the results shown in Figure 4, it is evident that regardless of the FCG pattern, the predictive performance of traditional single-input–single-output NN models (T0) exhibits significant dependence on the data volume. While model accuracy improves with an increase in sample points, substantial deviations persist between the extrapolated predictions and the actual measured curves. This remains true even when the training sample proportion increases to 70%, with the model prediction errors becoming particularly pronounced during the later stages of fatigue crack growth. Thus, it is evident that conventional methods are unable to overcome the poor extrapolation performance inherent in small-sample modeling. Although increasing the sample size could further enhance model accuracy, doing so is fundamentally at odds with the goal of accelerating testing through ML.

**Figure 5:**
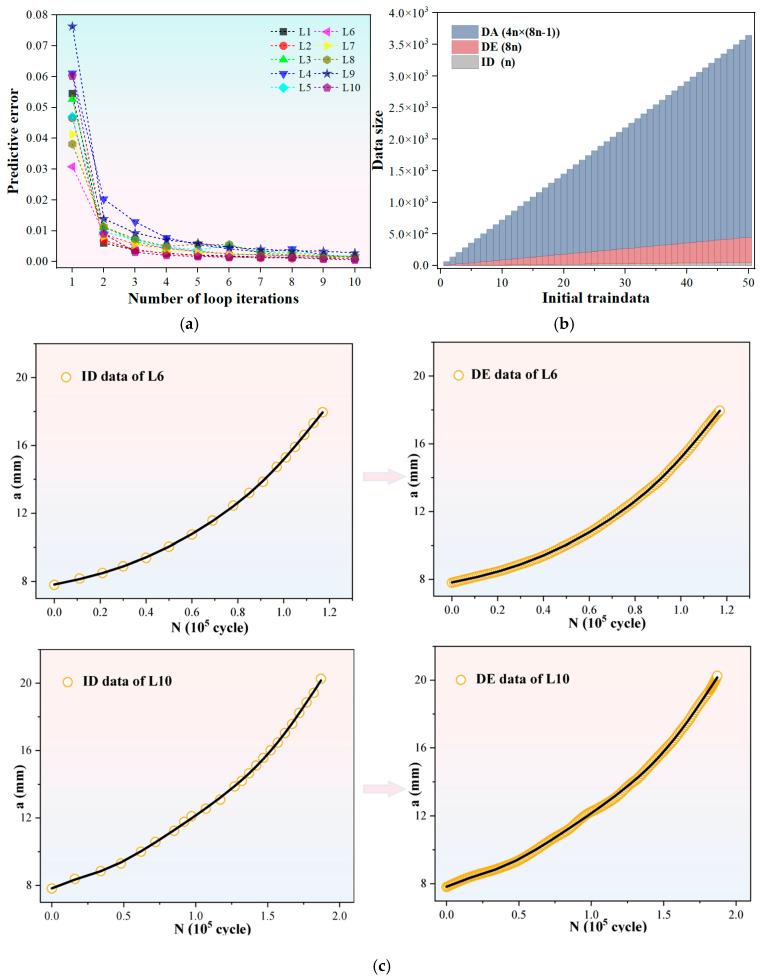
Data augmentation via interpolation-based model prediction. (**a**) Model error variance with increasing number of iterative cycles for the ten curves; (**b**) Exponential growth of data size after DE and DA; (**c**) Schematic comparison of data samples before and after data expansion for the ID of L6 and L10.

In order to compare the training efficiency with that of the complex-structured network model employed in the literature method [34], we also utilize the initial 50% of data points to train the FNN model. Specifically, the initial data sample (i.e., D_n_) is formed using the first 50% of data points from the experimental *a*-*N* curve, while the remaining half is reserved to serve as the validation set for assessing extrapolation performance. According to the workflow of the MLIES illustrated in Figure 1, we first employ the cyclical modeling approach to iteratively expand the ID. For each fatigue curve, the prediction accuracy of the NN models varies with the number of iterative cycles, as shown in Figure 5a. It can be observed that as the data expansion volume increases, the model error gradually decreases, indicating a positive impact on crack growth prediction. The most significant error reduction occurs at the initial expansion stage, suggesting that the interpolation prediction effectively extracts additional information from limited data, thereby improving model accuracy. Although error gradually decreases with more data, improvements plateau after threefold expansion and stabilize only at eightfold data expansion. Based on these results, an eightfold data expansion factor is established for the cyclic interpolation modeling.

Following iterative interpolation, the initial small data achieves multiple-fold expansion, increasing the data volume from n to 8n. Subsequently, following the workflow depicted in Figure 1 and based on the incremental information-driven DA methods outlined in Section 2, augmented datasets corresponding to each fatigue curve using strategies S1 and S2 were sequentially constructed. This approach not only enriched the data samples but also incorporated information such as the incremental growth and gradient of FCG into the NN model training. As shown in Figure 5b and Table 1, the two-step approach of data expansion and data augmentation enables exponential growth in small-sample datasets from ID to DE to DA.

### 3.2. Extrapolation Prediction of a-N Curve

**Figure 6:**
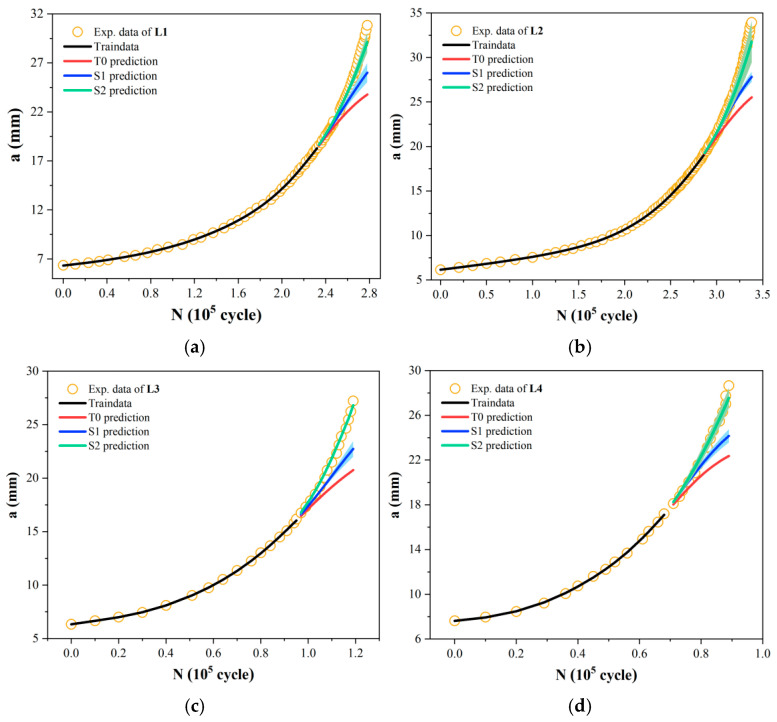

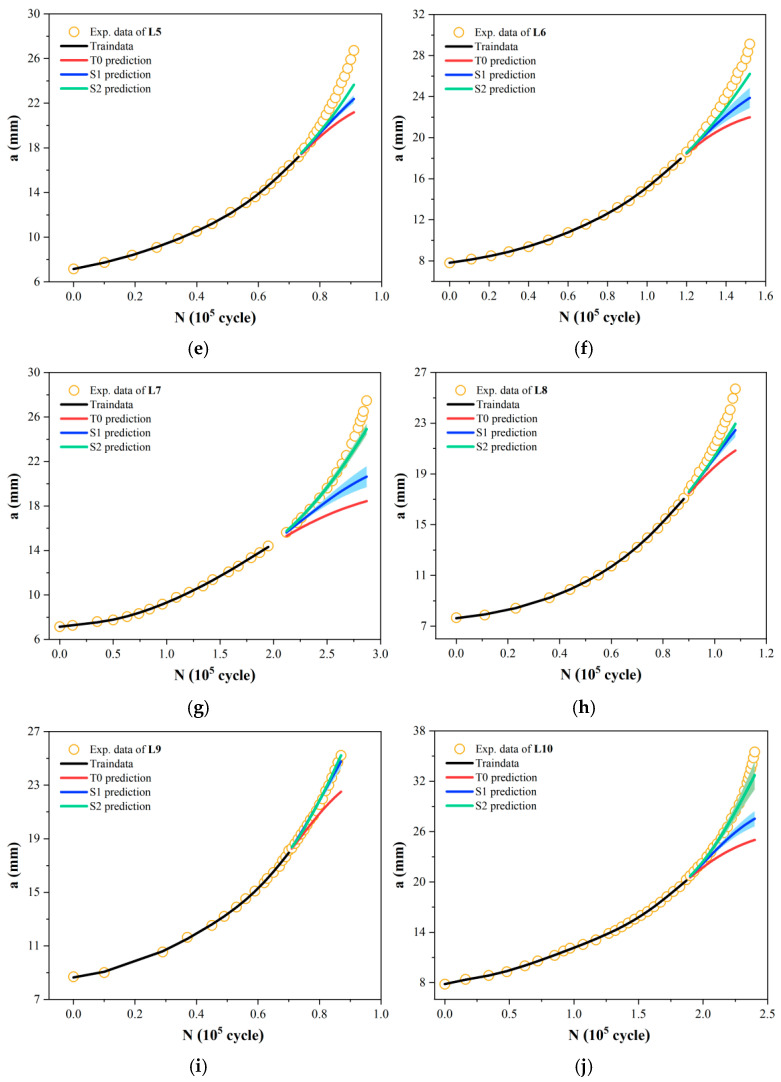
Prediction comparison of fatigue curve between the T0 model and the S1 and S2 models based on the proposed strategy. (**a**) L1; (**b**) L2; (**c**) L3; (**d**) L4; (**e**) L5; (**f**) L6; (**g**) L7; (**h**) L8; (**i**) L9; (**j**) L10.

As noted earlier, all networks use a simple single-hidden-layer architecture. The key hyperparameters to be optimized are the number of hidden nodes (ranging from 2 to 15) and the weight decay coefficient (with candidate values of 0.0005, 0.0007, and 0.001). Figure 6 shows the comparison of predictions used by a traditional T0 model and the two strategies of S1 and S2 for all the *a*-*N* curves under different experimental parameters. It can be seen that even though all the three models have not seen the second half of the data on the curve during training, the predictions of S1 and S2 models are still close to the experimental data since the ranges of predictions are relative small. Notably, the underlying principle governing titanium alloy is considered to differ from that of aluminum alloy. Thus, the results in Figure 6 indicate that the S1 and S2 models trained on the enhanced data possess great learning and extrapolating ability. IN contrast, the one input–one output T0 model trained on small data shows significant prediction deviation from the experimental results.

Our incremental information-based models seize the trend of crack growth with high accuracy. In particular, compared to the S1 strategy, the relative incremental information data used in S2 implicitly contains the underlying principles of crack evolution, thereby demonstrating a greater improvement in prediction accuracy—whether for aluminum alloys or titanium alloys. Furthermore, unlike the single crack prediction results of traditional T0 models, our strategy can provide a confidence interval for the predicted crack length based on the extrapolation method shown in Figure 2. Practically, in engineering applications, even if the materials are the same, there may be some errors in the processing technology, internal defects, and differences in material properties of the structure, resulting in changes in the fatigue crack growth rate law. This error caused by such uncertain factors will also accumulate with the crack growth process, reducing the accuracy of the prediction result. From this perspective, the confidence intervals this work provide for crack length prediction offer more meaningful reference results for subsequent crack growth analysis.

**Figure 7:**
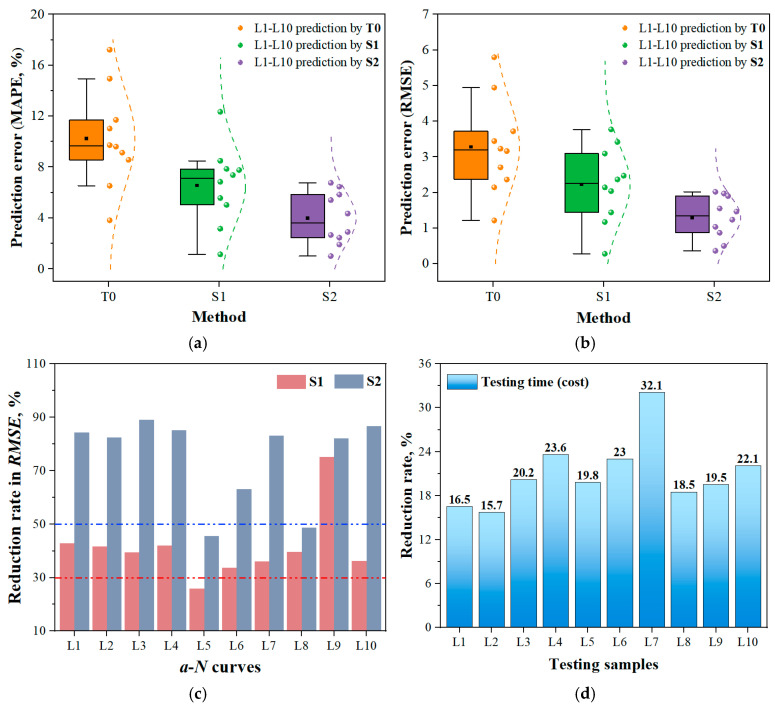
Prediction performance comparison of the proposed MLIES with the traditional T0 method. (**a**) MAPE; (**b**) RMSE; (**c**) Reduction rate of prediction error for S1 and S2 models compared to the T0 model; (**d**) Acceleration efficiency in fatigue test using the MLIES for each *a*-*N* curve.

Figure 7 illustrates the performance of the T0 method and the new strategy in extrapolation prediction across ten *a*-*N* curves. Two error metrics of mean absolute percentage error (*MAPE*) and root mean square error (*RMSE*) are used. From Figure 7a,b, it is evident that the model based on the S1 strategy exhibits significantly reduced prediction errors, indicating that the incorporation of absolute incremental information enhances the model’s learning of crack evolution characteristics. Compared to S1, under identical conditions of total data volume and training duration, the transition from incremental information to gradients-based incremental information further enhances the extrapolation prediction accuracy of the S2 model. Figure 7c presents the prediction error reduction rates of the S1 and S2 models compared to the T0 model. It can be seen that prediction errors vary across different curves. Considering differences in data volume and crack propagation patterns, such variations are expected. Overall, except for the L5 curve, the model based on the S1 strategy achieves over 30% improvement in prediction accuracy, while the S2 strategy-based model achieves over 50% improvement. This may be due to the sparse distribution of sample points in the early stages of the L5 fatigue test. The limited crack growth information contained in small samples increases the difficulty of subsequent predictions. Even so, the proposed ML approach still manages to improve prediction accuracy by over 20%, demonstrating its great learning and extrapolating ability.

### 3.3. Acceleration Efficiency of a-N Curve Testing

In the present work, the first 50% of the sample points in the fatigue test were used to train the NN model to predict the remaining portion of the *a*-*N* curve. Since each *a*-*N* curve has a different total cycle count, a standardized metric is needed to quantify the acceleration effect. The testing time/cost reduction rate was defined as (total cycle count-cycle count at 50% data)/total cycle count, i.e., (*N*_total_-*N*_50%_)/N_total_. The results of acceleration efficiency are shown in Figure 7d. It can be observed that for fatigue experiments involving different materials and parameters, the reduction in testing time/cost exceeds 15.5%, reaching up to 32.1%. This demonstrates that our method not only achieves precise prediction of fatigue test curves but also effectively enhances experimental efficiency, thus providing an alternative method to predict fatigue crack growth and accelerate the measurement of the *a*-*N* curve.

**Figure 8:**
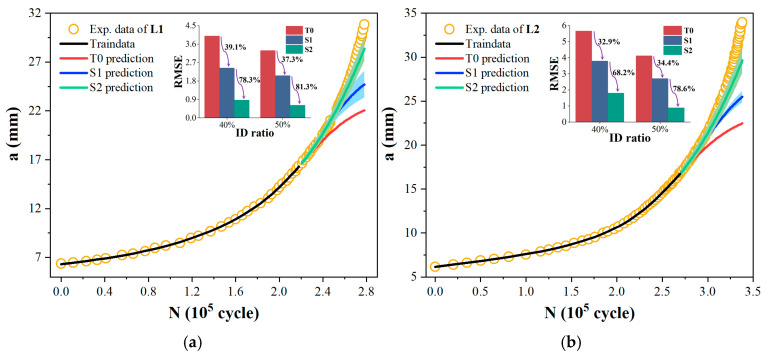
Prediction results of fatigue curves for the first 40% of sample points for modeling. (**a**) L1; (**b**) L2.

Additionally, to validate the stability of the MLIES, the training sample proportion was further reduced from 50% to 40% to predict the remaining portions of the *a*-*N* curve. L1 and L2 curves were selected as examples due to their representative data volumes among all the ten experimental curves, as shown in Table 1, with 40 and 17 data points, respectively—corresponding to larger and smaller sample sizes. The results are shown in Figure 8a,b. It can be observed that the traditional T0 model exhibits greater prediction bias. The prediction errors (RMSE) of the L1 and L2 curves based on the S2 increased from 0.613 and 0.881 with 50% data samples to 0.865 and 1.794, respectively. Nevertheless, compared to T0 model predictions, the S2 model trained on a reduced training sample size still achieved a reduction in prediction error exceeding 75%. Although the reduced initial data volume increased the prediction error of the S1 model, its accuracy remained over 30% higher than that of the T0 model with equivalent initial data. This demonstrates that the MLIES maintains robust predictive performance even with less initial data, highlighting its higher tolerance for small samples and greater stability in fatigue prediction accuracy.

Finally, it is worth emphasizing that the simple single-hidden-layer NN architecture adopted in our proposed MLIES significantly accelerates model training efficiency. For example, the total training time based on the NN model is roughly ten minutes on a server equipped with a 1.7 GHz Intel Core i5 (general PC configuration) when the number of the ID on *a*-*N* curve is 17 (such as L5~L9). This effectively bridges the gap between data-driven methods and real-time online monitoring/acceleration testing, translating methodological potential into tangible benefits for practical engineering applications.

Meanwhile, it should be noted that the current study provides a data-driven approach for *a*-*N* curve prediction and points a way forward for related work. In further research, we will also take into account practical engineering requirements, as well as the comparison and integration of relevant physics-based models with the data technique to promote fatigue crack analysis.

## 4. Conclusions

To accelerate fatigue testing and address the challenge of small-sample modeling in *a*-*N* curve prediction, we propose an MLIES that integrates cyclic modeling with interpolation-based sample expansion and incremental information modeling. By developing data enhancement methods based on absolute increments (S1) and relative increments (S2), along with their associated NN models, the proposed strategy achieves high-precision extrapolation predictions of fatigue crack growth.

Through validation on fatigue curve predictions for two typical materials—aluminum alloy and titanium alloy—our strategy demonstrates outstanding generalization and predictive performance. Compared to traditional single-input–single-output modeling approaches, the S1 model improves the prediction accuracy of fatigue curves by over 30%. The S2 strategy, which incorporates gradients-based incremental information, further enhances prediction accuracy by more than 50%. Moreover, by integrating a simple and rapidly responsive single-hidden-layer NN architecture, our approach enables fast modeling and responsive prediction, reducing experimental testing duration and costs by over 15%. This provides a strategic foundation for realizing online accelerated testing of fatigue curves.

Furthermore, the predictive method based on the MLIES is capable of providing confidence intervals for fatigue crack length predictions, thereby inherently capturing the uncertainty associated with the fatigue process. This aligns well with real-world fatigue testing conditions and can subsequently supply more meaningful data for risk-quantification-based analysis of fatigue crack propagation.

## Figures and Tables

**Table 1:** Fatigue curve information.

Alloys	Experimental Parameters			Fatigue Curve	Data Size in Curve	Data Size of ID	Data Size After DE	Data Size After DA
	*R*	*σ*_max_ (MPa)	*a*_0_ (mm)					
7B04-T6	0.5	54.9	6.35	L1	79	40	316	2520
7B04-T6	0.5	35.6	6.15	L2	29	15	116	920
7B04-T6	0.5	60.5	6.34	L3	114	57	456	3640
7B04-T6	0.06	39.2	7.63	L4	33	17	132	1048
7B04-T6	0.5	59.4	7.16	L5	31	16	124	984
7B04-T6	0.06	39.2	7.80	L6	34	17	136	1080
TA15	0.5	115.9	7.15	L7	34	17	136	1080
TA15	0.06	103.3	7.67	L8	33	17	132	1048
TA15	0.06	103.3	8.70	L9	33	17	132	1048
TA15	−1	63.8	7.81	L10	47	24	188	1496

Notes: ID—the initial data points used for NN modeling, DE—data expansion, DA—data augmentation.

## Data Availability

The original contributions presented in this study are included in the article. Further inquiries can be directed to the corresponding author.

## References

[1] Davies K., Feddersen C. (1973). Evaluation of fatigue-crack growth rates by polynomial curve fitting. Int. J. Fract..

[2] Liu H. (1963). Fatigue crack propagation and applied stress range—An energy approach. J. Basic Eng..

[3] Panasyuk V., Ratych L., Dmytrakh I. (1984). Fatigue crack growth in corrosive environments. Fatigue Fract. Eng. Mater. Struct..

[4] Mu Z., Chen D., Zhu Z., Ding W., Tian S. (2013). Fatigue Crack Growth Behavior of Aerospace Aluminum Alloy LD2 Under Corrosion. Acta Aeronaut. Astronaut. Sin..

[5] Frost N., Dugdale D. (1958). The propagation of fatigue cracks in sheet specimens. J. Mech. Phys. Solids.

[6] Frost N., Marsh K., Pook L. (1999). Metal Fatigue.

[7] Amsterdam E., Wiegman J.W.E., Nawijn M., De Hosson J.T.M. (2022). The effect of crack length and maximum stress on the fatigue crack growth rates of engineering alloys. Int. J. Fatigue.

[8] Ni C., Hua L., Wang X., Wang Z., Ma Z. (2018). Numerical and experimental method for the prediction of the propagation life of fatigue crack on metallic materials. J. Mech. Sci. Technol..

[9] De Iorio A., Grasso M., Penta F., Pucillo G.P. (2012). A three-parameter model for fatigue crack growth data analysis. Fract. Struct. Integr..

[10] Novikov Y., Zoteev V., Gudkov A. (1976). Propagation of a fatigue crack in a weld. Strength Mater..

[11] Chen J., Liu Y. (2022). Fatigue modeling using neural networks: A comprehensive review. Fatigue Fract. Eng. Mater. Struct..

[12] Wei X., Zhang C., Han S., Jia Z., Wang C., Xu W. (2022). High cycle fatigue S-N curve prediction of steels based on transfer learning guided long short term memory network. Int. J. Fatigue.

[13] Li S.G., Chen Q.R., Huang L., Chen M., Wei C.D., Yue Z.J., Liu R.X., Tong C., Liu Q. (2024). Data-driven approach to predict the fatigue properties of ferrous metal materials using the cGAN and machine-learning algorithms. Adv. Manuf..

[14] Zhan Z., Zhang M., He X., Li X., Wang Z., Chen X., Han B., Hu W., Meng Q., Li H. (2025). Advances in machine learning for predicting fatigue behavior: From material properties to fatigue life and fatigue crack growth. Int. J. Struct. Integr..

[15] Lei Z., Zhou J., Wang Y., Chen S., Zhang Y., Zhang Z. (2025). A Multi-Source Data-Driven Machine learning framework for predicting fatigue crack paths in polycrystalline superalloys. Int. J. Fatigue.

[16] Tan Z.X., Thambiratnam D.P., Chan T.H.T., Razak H.A. (2017). Detecting damage in steel beams using modal strain energy based damage index and Artificial Neural Network. Eng. Fail. Anal..

[17] Shamsirband S., Khansari N. (2021). Micro-mechanical damage diagnosis methodologies based on machine learning and deep learning models. J. Zhejiang Univ. Sci. A.

[18] Huang Z., Yan J., Zhang J., Han C., Peng J., Cheng J., Wang Z., Luo M., Yin P. (2024). Deep Learning-Based Fatigue Strength Prediction for Ferrous Alloy. Processes.

[19] Fan J., Wang Z., Liu C., Shi D., Yang X. (2024). A tensile properties-related fatigue strength predicted machine learning framework for alloys used in aerospace. Eng. Fract. Mech..

[20] Wang H., Li B., Gong J., Xuan F.Z. (2023). Machine learning-based fatigue life prediction of metal materials: Perspectives of physics-informed and data-driven hybrid methods. Eng. Fract. Mech..

[21] Cao F., Tao S., Chen R., Huang S., He G., Li W., Liu Z. (2025). Fatigue crack growth rate models of Ti-6Al-4V alloy considering temperature and stress ratio. J. Mech. Sci. Technol..

[22] Zhang C., Hu Z., Zhang W., Zhuang X., Zhao Z. (2025). Machine learning assisted calibration of a fatigue crack growth model considering temperature and stress ratio conditions. Eng. Fract. Mech..

[23] Hidetoshi F., Mackay D., Bhadeshia H. (1996). Bayesian Neural Network Analysis of Fatigue Crack Growth Rate in Nickel Base Superalloys. ISIJ Int..

[24] Lee J.H., Lee H.Y., Hong S.G., Lee S.B. (2017). Fatigue crack growth behavior of Mod.9Cr-1Mo steel at elevated temperatures: Effect of temperature, loading frequency and R ratio. J. Mech. Sci. Technol..

[25] Ji Y., Wu S. (2018). Effect of Stress Ratio on Fatigue Crack Growth Behavior of Ti-6Al-2Zr-1Mo-1V Alloy. JAM.

[26] Ye J., Cui P., Guo W. (2025). A machine learning-based method for fatigue crack growth rate prediction in the near-threshold region. Eng. Fract. Mech..

[27] Fotovati A., Goswami T. (2004). Prediction of elevated temperature fatigue crack growth rates in TI-6AL-4V alloy-neural network approach. Mater. Des..

[28] Zhang W., Bao Z., Jiang S., He J. (2016). An Artificial Neural Network-Based Algorithm for Evaluation of Fatigue Crack Propagation Considering Nonlinear Damage Accumulation. Materials.

[29] Mohanty J.R., Verma B.B., Ray P.K., Parhi D.R. (2010). Application of Artificial Neural Network for Fatigue Life Prediction under Interspersed Mode-I Spike Overload. J. Test. Eval..

[30] Pidaparti R., Palakal M. (1995). Neural network approach to fatigue-crack-growth predictions under aircraft spectrum loadings. J. Aircr..

[31] Okafor A.C., Singh N., Singh N., Oguejiofor B.N. (2016). Acoustic emission detection and prediction of fatigue crack propagation in composite patch repairs using neural network. J. Thermoplast. Compos. Mater..

[32] Nechval K.N., Nechval N.A., Bausova I., Skiltere D., Strelchonok V.F. (2014). Prediction of Fatigue Crack Growth Process via Artificial Neural Network Technique. Int. J. Comput..

[33] Haynes R., Joshi G., Bradley N. Machine Learning Based Prognostics of Fatigue Crack Growth in Notch Pre-cracked Aluminum 7075-T6 Rivet Hole. Proceedings of the Annual Conference of the Prognostics and Health Management Society.

[34] Ma X., He X., Tu Z. (2021). Prediction of fatigue–crack growth with neural network-based increment learning scheme. Eng. Fract. Mech..

